# Predicting cross-protection against foot-and-mouth disease virus strains by serology after vaccination

**DOI:** 10.3389/fvets.2022.1027006

**Published:** 2022-12-01

**Authors:** Simon Gubbins, David J. Paton, Aldo Dekker, Anna B. Ludi, Ginette Wilsden, Clare F. J. Browning, Michael Eschbaumer, Jamie Barnabei, Hernando Duque, Lisa L. Pauszek, Donald P. King

**Affiliations:** ^1^The Pirbright Institute, Pirbright, United Kingdom; ^2^Laboratory Vesicular Diseases, Department of Virology and Molecular Biology, Wageningen Bioveterinary Research, Lelystad, Netherlands; ^3^Institute of Diagnostic Virology, Friedrich-Loeffler-Institut, Greifswald, Germany; ^4^Plum Island Animal Disease Center, Greenport, NY, United States

**Keywords:** foot-and-mouth disease, vaccination, predicting cross-protection, immune correlate, serology, virus neutralising antibody, post-vaccination monitoring

## Abstract

Serology is widely used to predict whether vaccinated individuals and populations will be protected against infectious diseases, including foot-and-mouth disease (FMD), which affects cloven-hoofed animals. Neutralising antibody titres to FMD challenge viruses correlate to protection against FMD, for vaccinated cattle that are infected with the same strain as in the vaccine (homologous protection). Similar relationships exist for cross-strain protection between different vaccine and challenge viruses, although much less data are available for these heterologous studies. Poor inter-laboratory reproducibility of the virus neutralisation test (VNT) also hampers comparisons between studies. Therefore, day-of-challenge sera (*n* = 180) were assembled from 13 previous FMD cross-protection experiments for serotypes O (*n* = 2), A (*n* = 10), and SAT 2 (*n* = 1). These were tested by VNT against the challenge viruses at the FMD FAO World Reference Laboratory (WRLFMD) and the titres were compared to challenge outcomes (protected or not). This dataset was combined with equivalent serology and protection data for 61 sera from four cross-protection experiments carried out at WRLFMD for serotypes O (*n* = 2), A (*n* = 1), and Asia 1 (*n* = 1). VNT results and protection outcomes were also analysed for a serotype O cross-protection experiment involving 39 cattle, where the sera were not available for retesting at WRLFMD. Three categories of association between heterologous neutralising antibody titre and heterologous protection were found (Group 1–3). The log_10_ reciprocal titres associated on average with 75% protection (with 95% credible limits) were: Group 1: 2.46 (2.11–2.97); Group 2: 1.67 (1.49–1.92); Group 3: 1.17 (1.06–1.30). Further cross-protection data are needed to understand the factors that underpin this variability and to develop more robust antibody thresholds. Establishing cut-off serological titres that can be used to score the adequacy of vaccine-induced immunity will facilitate the monitoring and thereby the performance of FMD vaccination in the field.

## Introduction

Foot-and-mouth disease (FMD), which affects domestic and free-living ungulates, is a vesicular disease caused by an RNA virus (FMDV) in the family *Picornaviridae*, genus *Aphthovirus*. The virus is contagious and antigenically diverse, with six currently circulating serotypes (1) that do not cross-protect and multiple strains within serotypes that cross-protect to variable degrees. Consequently, infection and reinfection can be common in endemic settings and the virus may be reintroduced into countries where it has been eliminated. Vaccines are an important control option for both prophylactic and reactive responses to FMD (2).

Current vaccines are produced from inactivated cell culture grown virus capsids formulated with an oil or aqueous adjuvant. The protection afforded by FMD vaccines is relatively short-lived and may be strain dependent but can be strengthened and prolonged by increasing the vaccine's potency and by giving boosters, which will also improve the antigenic coverage of field strains but is more expensive. Before a new vaccine strain can be registered, a potency test is normally conducted by vaccinating target hosts (usually cattle, sometimes pigs) and challenging them 21–28 days later with FMDV that is the same as (homologous) to the vaccine strain. In cattle, the test involves inoculation of FMDV into the tongue and if the challenge virus is blocked from generalisation to cause vesicles on the feet, then the animal is considered protected (3). Antibodies are a major component of acquired immunity and once a correlation can be shown between protection and day-of-challenge antibody titre, then serology can be used as an indirect potency test for acceptance of subsequent vaccine batches without challenge [batch release testing (3)]. The antigenic suitability of a vaccine strain can be assessed serologically by comparing the antibody titres of sera from vaccinated animals against the vaccine strain and one or more relevant field strains. Vaccine selection is informed by this combination of verification of homologous potency and antigenic match, but there is uncertainty in how these two factors interact. A heterologous potency test that takes account of both potency and match is likely to be a better predictor of vaccine performance in the field but is laborious, expensive, and unethical for routine use. An indirect heterologous potency test could be based on heterologous serology, without a prior challenge test, if it could be shown that the titres associated with protection do not differ between strains. A study of heterologous protection with challenge for several antigenically distinct serotype A strains showed a better correlation between protection and day-of-challenge neutralising antibody titre to the challenge strain than between protection and titre to the vaccine strain (4). High-potency vaccines that elicit strong antibody responses were found to protect even against strains for which there was a poor antigenic match. Since there is poor reproducibility of virus neutralisation tests (VNT) between laboratories (5), this study attempted to quantify the variation in the titres associated with cross-protection when all of the serology was performed in a single laboratory (the World Reference Laboratory for FMD, WRLFMD, at the Pirbright Institute). Other aims were to consider if (1) the titres associated with protection after homologous challenge would be equivalent to those after heterologous challenge, provided that the heterologous virus was used in the VNT; and if (2) the titres associated with protection are not affected by boosting.

## Materials and methods

### Protection studies

Day-of-challenge sera (*n* = 241) were obtained or had already been tested from 17 cross-protection experiments with 245 cattle (four sera unavailable) and four serotypes [O, A, Asia 1, and Southern African Territories 2 (SAT 2)] carried out under high containment between 2007 and 2020 in Germany, the USA, the Netherlands, and the UK (Table 1). The vaccine strains and challenge viruses had been isolated between 1964 and 2015 originating from the Middle East, North Africa and South America. The cattle used were conventionally reared, of various breeds and mostly between 6 and 12 months of age. Most of the vaccines had been supplied by Merial/Boehringer Ingelheim, formulated at a potency of at least 6 PD50/dose, from antigen banks maintained by FMD-free countries and given as monovalent full or reduced-volume doses. Only in the eight experiments of Brehm et al. (4) had potency tests been performed to establish the homologous potency of the same vaccines also used to study cross-protection. Most of the vaccines were double oil emulsion (DOE) formulations that were administered intramuscularly. In the SAT 2 study, vaccination was by the subcutaneous route. In one study (9), an aqueous multivalent vaccine with a saponin adjuvant that had not been formulated from bank antigen was given subcutaneously. Another study (8) employed a multivalent vaccine from Vecol in South America, with a serotype O and a serotype A component. The studies were carried out to test the ability of vaccines to protect against challenge viruses that had an incomplete antigenic match [one-way relationship *r*_1_ values of 0–0.64; Rweyemamu (11)] to the vaccine strain in question.

**Table 1:** Summary of heterologous vaccination-challenge studies for foot-and-mouth disease included in the analysis.

**Expt**	**Serotype**	**Vaccine strain**	**Challenge virus (strain)**	***r_1_-*value**	Number challenged^*^	**Number protected**	**Serology test at WRLFMD**	**Mean log_10_ VNT at challenge^†^1**	**References**
1	O	O Manisa	O/ALG/3/2014 (O/ME-SA/Ind-2001)	0.13	15	7	2015	2.31	(6)
2	O	O Manisa	O Campos	0.6	39	20	–	– (1.82)	(7)
3	O	O Manisa	O/IRN/34/2006 (O/ME-SA/PanAsia2)	0.64	15	7	2007	1.41	Pirbright Institute unpublished
4	O	O Campos^‡^	O/Orellana-036/ Ecuador 2010^**^ (O/EURO-SA/unnamed)	0.24	10	9	2021	2.06	(8)
5	O	O Campos^‡^	O/Orellana-036/ Ecuador 2010 (O/EURO-SA/unnamed)	0.16	10	5	2021	1.40	(8)
6	A	A Iran 05/A Sau 95^‡^	A/IRN/22/2015 (A/Asia/G/VII)	0/0.25	16	9	2016	1.25	(9)
7	A	A22 Iraq	A/IRN/22/2015 (A/ASIA/GVII)	0.2	7	2	2017	1.26 (1.16)	(10)
8	A	A May 97	A/IRN/22/2015 (A/ASIA/GVII)	ND	22	18	2017/18	1.45 (1.33)	(10)
9	A	A22 Iraq	A Iran 96	0.09	15	9	2020	0.98	(4)
10	A	A22 Iraq	A Egypt 06	0.12	15^††^	11	2020	1.43	(4)
11	A	A22 Iraq	A Iran 99	0.04	15^‡‡^	7	2020	1.21	(4)
12	A	A Iran 99	A22 Iraq	0.10	15^††^	12	2020	1.23	(4)
13	A	A Iran 99	A Iran 96	0.23	15	13	2020	1.43	(4)
14	A	A Iran 96	A Iran 99	0.12	15	11	2020	1.04	(4)
15	A	A Iran 96	A22 Iraq	0.12	15^‡‡^	5	2020	0.86	(4)
16	A	A Iran 96	A22 Iraq	0.10	15	10	2020	1.21	(4)
17	Asia 1	Asia 1 Shamir	Asia 1/TUR/49/11 (Asia 1/ASIA/Sindh-08)	0.20	15	13	2012	1.40	Pirbright Institute unpublished
18	SAT 2	SAT 2 Sau 2000	SAT 2/LIB/40/2012 (SAT 2/VII/unnamed)	ND	15	11	2020	1.60 (1.11)	Dekker et al. unpublished

* Cattle challenged at 21 days post vaccination.

† VNT, virus neutralisation titre; titres shown out with brackets are those obtained at WRLFMD, while those within brackets are those obtained at the original laboratory; means were calculated for all cattle in the study regardless of whether or not they were protected.

‡ Multivalent vaccine containing other serotypes.

** Cattle boosted at 14 days post first vaccination and challenged at 21 days post booster vaccination.

†† Serum from one protected animal in the original study no longer available.

‡‡ Serum from one unprotected animal in the original study no longer available.

All of the challenges were by tongue inoculation of 10^4^ bovine 50% infectious doses of virus, or an equivalent based on titration in cell culture (12). Of the cattle, 159 (65%) were protected by vaccination from virus generalisation to the feet, whereas 86 (35%) cattle were unprotected. The sera had been collected at 21 days post vaccination (dpv), but in one study, 10 cattle were boosted at 14 dpv and then challenged 21 days after this second vaccination (8).

VNT results from an 18th study of cross-protection were also included in the analysis (Table 1, experiment 2). In this study, 39 cattle had been immunised with a DOE formulation of O Manisa vaccine produced by Indian Immunologicals Ltd. Only twenty of the cattle (51%) had been protected from virus generalisation after challenge with O Campos despite earlier serology showing a relatively good antigenic match to O Manisa [*r*_1_ = 0.6; (7)]. These 39 sera were not available for retesting by VNT at WRLFMD.

### Virus neutralisation test

Archived sera (*n* = 180) were shipped to WRLFMD and tested with their in-house method, which follows the description in the WOAH Manual (3), using doubling final dilutions from 0.9 log_10_ to 3.0 log_10_, against the strains used for challenge in the respective cross-protection studies. In the case of O Ecuador 2010, the viruses used in the challenge and serology had been isolated from different but contemporaneous and epidemiologically linked outbreaks. The titre of the virus and of the positive control serum were controlled by reference to their running mean values and the Kärber method was used for titre calculation (13). For analysis, titres of <0.9 log_10_ were scored as 0.8. Sera collected from experiments at WRLFMD (*n* = 61, Table 1: 1, 3, 6, 17) had been tested according to this method between 2007 and 2021.

### Statistical methods

A logistic regression model was used to relate the probability of protection to VNT. Specifically, the probability (*p*) that an animal with a titre log_10_ *T* was protected after challenge was given by log[*p*/(1–*p*)] = *a* + *b*log_10_*T*, where *a* is the intercept and *b* is the slope. To explore how the level of protection for a given titre varies amongst serotypes and strains, three possibilities were considered for slope and intercept: (i) they are independent of strain/serotype; (ii) they differ amongst serotypes but are common within a serotype; and (iii) they differ amongst strains, including within a serotype. Variation amongst serotypes or strains was incorporated by including hierarchical structure in the parameters so that the parameters for serotype/strain j are drawn from higher-order normal distributions, so that *a*_*j*_ ~ N(μ_*a*_,σ_*a*_) and *b*_*j*_ ~ N(μ_*b*_,σ_*b*_), where the μs and σs are the means and standard deviations. A total of nine models was considered (Supplementary Table 1).

Parameters were estimated in a Bayesian framework. A Bernoulli likelihood was assumed for the data (i.e., whether an animal was protected or not). Here protection was defined based on the development of lesions on the feet: if no lesions developed the animal was considered protected; if lesions developed at least one foot, it was considered to not be protected. Diffuse normal priors (with mean 0 and standard deviation 10) were assumed for *a* and *b* (in a non-hierarchical model) or μ_*a*_ or μ_*b*_ (in the hierarchical model). Diffuse exponential priors (with mean 100) were assumed for σ_*a*_ or σ_*b*_ in the hierarchical models. The methods were implemented using OpenBUGS (version 3.2.3; https://www.mrc-bsu.cam.ac.uk/software/bugs/openbugs/). Two chains each of 120,000 samples were run, with the first 20,000 iterations discarded to allow for burn-in of the chain. Chains were subsequently thinned by selecting every tenth iteration to reduce autocorrelation amongst the samples. Convergence of the chains was monitored visually and using the Gelman-Rubin statistic in OpenBUGS. Different models for the variation amongst serotypes/strains in parameters were compared using the deviance information criterion (DIC) (14).

Three analyses were conducted using the approach outlined above. First, the results from all studies in Table 1 were included in the analysis. Second, the results from all studies in Table 1 except those of Nagendrakumar et al. (7) were included in the analysis, to test the sensitivity of the results to the one study for which sera were not retested at WRLFMD. Finally, for the three studies where the titre results from the original laboratory were available for each animal [studies 7, 8, and 18; Wageningen BioVeterinary Research (WBVR)] the effect of using these titres was also analysed.

## Results

**Figure 1:**
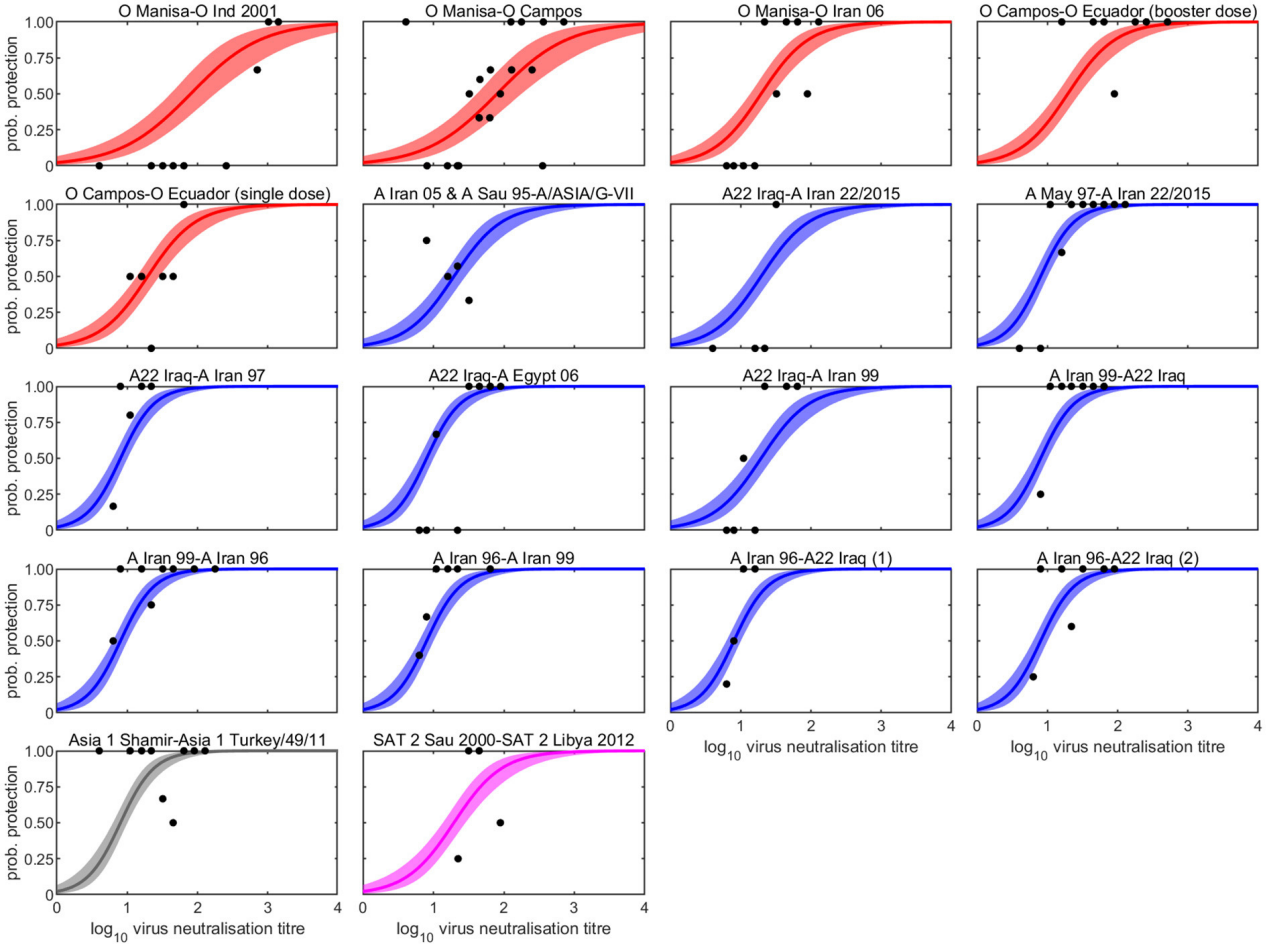
Observed and estimated probability that a vaccinated bovine animal is protected following heterologous challenge and its dependence on log_10_ virus neutralisation titre for eighteen vaccine-challenge studies. The vaccine and challenge strains are identified before and after the hyphen, respectively. Each plot shows the observed proportion of cattle protected at each titre in the study (circles) and the posterior median (line) and 95% credible interval (shading) for the probability of protection. Colour indicates serotype: O (red), A (blue), Asia 1 (grey), and SAT 2 (magenta).

**Figure 2:**
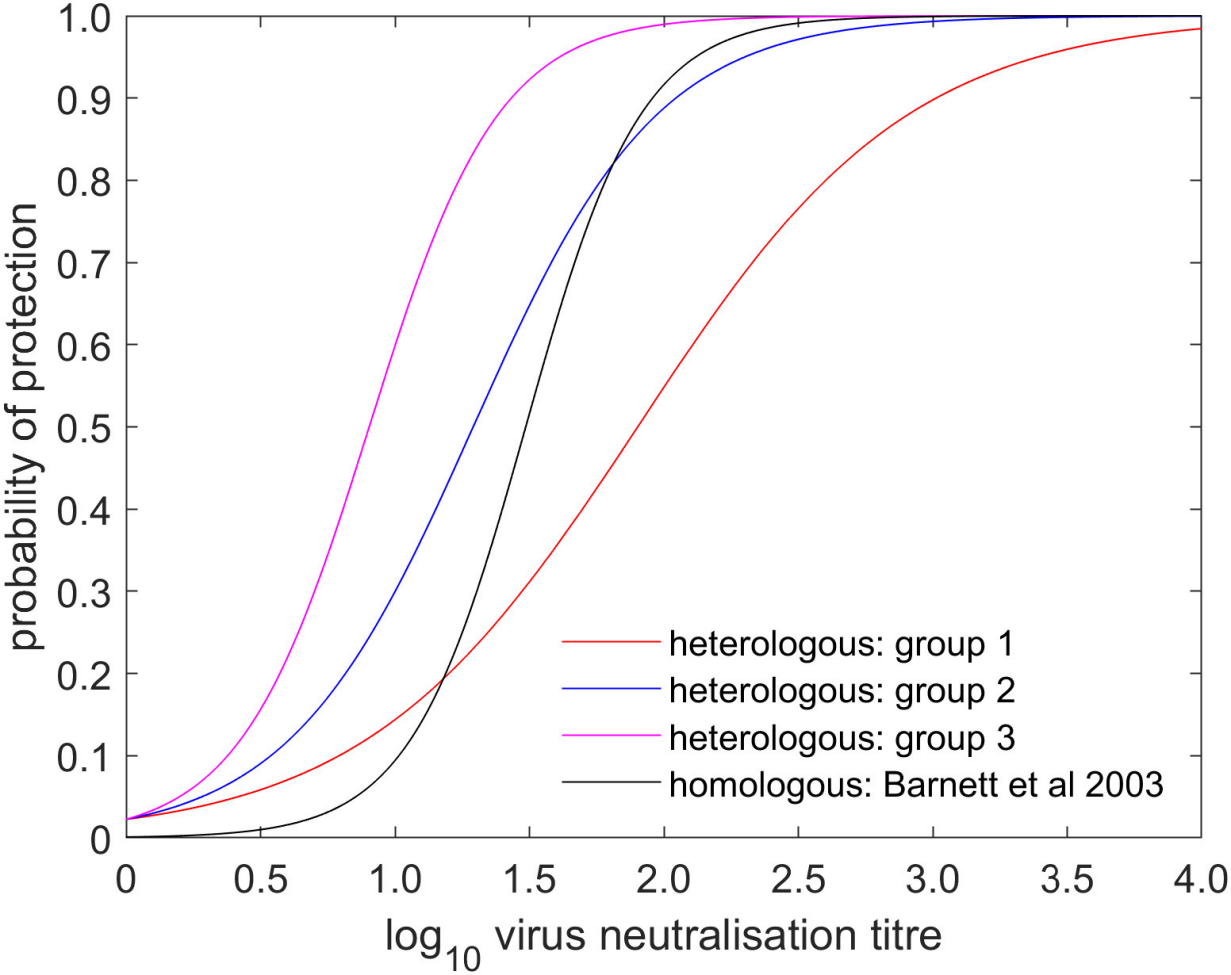
Estimated probability that a vaccinated bovine animal is protected following challenge and its dependence on log_10_ virus neutralisation titre. The plot shows the posterior median for the probability of heterologous protection for group 1 (comprising experiments 1 & 2; red), group 2 (comprising experiments 3, 4, 5, 6, 7, 11, & 18; blue) and group 3 (comprising experiments 8, 9, 10, 12, 13, 14, 15, 16, & 17; magenta) and the probability of homologous protection as estimated by Barnett et al. (5) (black).

The serology results and protection outcomes for each animal are available as Supplementary material 1. The probability of protection was best captured by a model in which the intercept was common to all studies and the slope varied amongst studies (Supplementary Table 1). However, *post-hoc* comparison of the slopes suggested the experiments could be divided into three groups: group 1 comprising experiments 1 and 2 (two O Manisa vaccine studies); group 2 comprising experiments 3, 4, 5, 6, 7, 11, 18 (serotypes O, A, and SAT 2 and also including one O Manisa study), and group 3 comprising experiments 8, 9, 10, 12, 13, 14, 15, 16, and 17 (serotypes A and Asia 1), which did indeed yield a much better fit to the data (Supplementary Table 1). In experiment 4, in which O Campos vaccinated cattle were boosted prior to challenge, the boost did not change the relationship between VN titre and cross-protection compared to single vaccination (Experiment 5), both being categorised as Group 2. The observed proportions of protected cattle and the fitted curves for probability of protection are shown for each experiment in Figure 1. In addition, the expected probabilities of protection for the three groups are shown in Figure 2 and Table 2 and the corresponding estimates for the intercept (*a*) and slopes (*b*) are provided in Supplementary Table 2.

**Table 2:** Estimated log_10_ virus neutralisation titres at which vaccinated cattle are protected from challenge with foot-and-mouth disease virus.

	**Log**_**10**_ **50% protective titre**			**Log**_**10**_ **75% protective titre**			**Log**_**10**_ **95% protective titre**		
	Estimate^*^	**95% credible limits**		**Estimate**	**95% credible limits**		**Estimate**	**95% credible limits**	
		**Lower**	**Upper**		**Lower**	**Upper**		**Lower**	**Upper**
Group 1^†^	1.90	1.61	2.25	2.46	2.11	2.97	3.39	2.86	4.29
Group 2^†^	1.29	1.13	1.46	1.67	1.49	1.92	2.30	2.01	2.77
Group 3^†^	0.90	0.80	1.00	1.17	1.06	1.30	1.61	1.43	1.87
Homologous protection^‡^	1.49	1.38	1.58	–	–	–	2.12	–	–

* Posterior median.

† Group 1 comprises experiments 1 & 2; group 2 comprises experiments 3, 4, 5, 6, 7, 11, & 18; and group 3 comprises experiments 8, 9, 10, 12, 13, 14, 15, 16, & 17.

‡ From Barnett et al. (5), their Table 2.

**Figure 3:**
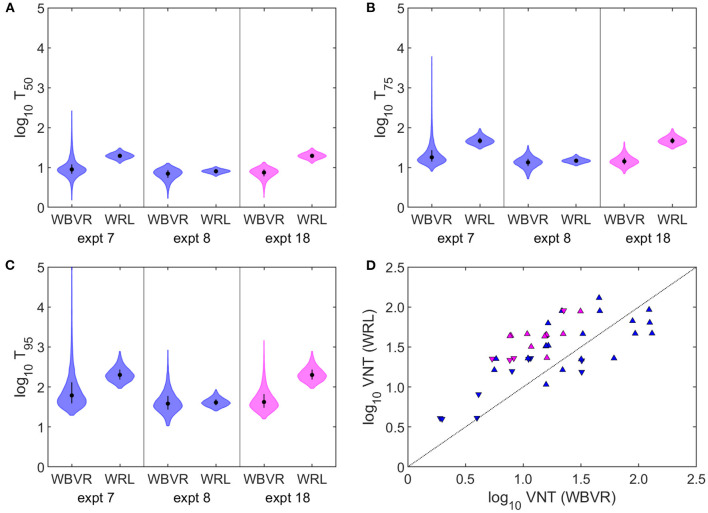
Impact of inter-laboratory variation in virus neutralisation titres (VNT) on estimates of titres required for protection. **(A–C)** VNT required for **(A)** 50%, **(B)** 75%, or **(C)** 95% of cattle to be protected from challenge with foot-and-mouth disease virus estimated using VNT obtained at WBVR or WRLFMD. Violin plots show the median (circle), interquartile range (error bar), and density (shape) for the posterior distribution. **(D)** VNT results obtained at WBVR and those obtained at WRLFMD in this study. Symbols indicate protection status of the animal: up-triangle (protected), down-triangle (not protected). The dotted line indicates equality. In all panels colour indicates serotype: A (blue) and SAT 2 (magenta). Details of the three experiments (7, 8, and 18) are shown in Table 1.

The best-fitting model was not influenced by the inclusion of the experiment for which the sera could not be retested at WRLFMD (7) (Supplementary Table 1). Furthermore, the same best-fitting model was selected when the results of experiments 7, 8, and 18 were analysed using the titres obtained at the original laboratory (Supplementary Table 1). However, the estimated titres required for protection were lower using the original WBVR titres compared with those obtained using the WRLFMD titres (Figures 3A–C). This reflects the fact that the titres obtained by WRLFMD were typically higher than those obtained by WBVR (Figure 3D).

## Discussion

Antibody levels, often measured by VNT, are widely used predictors of protection against FMD in vaccinated animals (3, 15). Our understanding about the levels of antibody, or other immune responses (16) that are associated with protection is mainly derived from homologous potency tests in which the same virus strain is used in both the vaccine and the post-vaccination challenge. In reality, FMD vaccination must protect against field viruses that belong to the same serotype but may be antigenically different (i.e., are heterologous) from the vaccine strains to variable extents. A simple approach to assess cross-protection is to measure the amount of antibody that vaccinated animals have against the field virus of concern. This takes account of both vaccine potency and regime and antigenic suitability as well as avoiding the need to obtain proprietary vaccine strains from vaccine producers. It also has the advantage of not requiring antiserum to a monovalent vaccine, making it applicable to animals vaccinated with multiple strains of a given serotype. However, the adoption of a common heterologous serological threshold of protection will be difficult if results for different vaccine/challenge combinations are highly variable unless such variability can be controlled for. To explore this, the present study examined the correlation between day-of-challenge antibody titres to heterologous challenge viruses and the challenge outcomes. The VNT was used for serology because of the recognised correlation between neutralising antibodies and protection, and because, unlike ELISA systems, it is relatively easy to change the virus used in the test to match the threat in the field. To minimise reproducibility problems when VNT is performed in different laboratories, the sera were all tested in one place. Curves relating neutralising antibody levels to the probability of protection were established and analysed from 18 previously performed cross-protection studies with four FMDV serotypes.

The WOAH minimum standard for FMD vaccines is three 50% protective doses (PD_50_) per full dose. For oil emulsion vaccines, this equates to an ~71% probability of protection (17). In the present study, the average heterologous neutralising antibody titre associated with 75% protection ranged from 1.17 to 2.46 log_10_, so it was not possible to define a common threshold for all the vaccine/challenge virus combinations. Three groups were defined, but with a larger dataset either additional groups or even a continuum of results might be anticipated. In Group 1 (experiments 1 and 2), involving different challenges of O Manisa vaccinated cattle, the highest antibody levels were required for protection. The results of the other experiments fell into two groups with 75% protection thresholds of 1.17 and 1.67. It is not obvious what determines the variable antibody thresholds for protection for different vaccine/challenge combinations and this requires further study. Possible explanatory variables include virus, vaccine, host, sample and test related factors. Serotype O studies were categorised in the groups with higher thresholds (Groups 1 & 2) and were the only serotype represented in the highest threshold group (Group 1). The serotype A studies, which were the most numerous, were evenly split between Groups 2 and 3. The single SAT 2 and Asia 1 studies were assigned to Groups 2 and 3, respectively. Strain-specific effects are not obvious, as experiments with O Manisa vaccine fall into Groups 1 and 2, and others with A22 vaccine fall into Groups 2 and 3. Similarly, use of the same challenge strain (A Iran 99) was associated with different thresholds (Groups 2 and 3). As most of the vaccines were produced as double oil emulsions by the same company, differences in formulation do not explain the variations in antibody thresholds for protection, although batch-specific differences might have had some impact. The three correlation groups also did not appear to be explained by the extent of antigenic difference between the vaccine and challenge strains (Table 1). Genetic differences between the cattle used might explain differences in their immune responses and the nature of their immune protection. It cannot be excluded that different passage histories of the FMD viruses used for cattle challenge and in the VNT might have resulted in antigenic changes that affected the relationship between *in vivo* and *in vitro* cross-protection. Furthermore, differences in virus strain growth characteristics in cell culture could affect the VNT and alter the relationship between *in vitro* and *in vivo* protection. Given the extended time over which the VNTs were performed, and the range of virus stocks used, a completely standardised test is unlikely to be achieved (due to variations in virus integrity, cell susceptibility, etc), even with testing at one location. Inter-laboratory variability of VNT results was not systematically analysed, but differences between WRLFMD and WBVR results were noted.

FMD cross-protection studies in livestock are infrequent. Brehm et al. (4) studied cross-protection for 8 different vaccine-challenge combinations, but most reports of such studies in cattle have been of small numbers or singleton experiments (6–8, 10, 18, 19). In contrast, homologous protection studies are performed as part of vaccine licencing and, over the last 40 years, many day-of-challenge sera from these have been analysed by VNT or ELISA and the results compared to protection outcomes (5, 17, 20–24). Barnett et al. (5) included an analysis of 246 sera collected 21 days post-vaccination from cattle vaccinated with serotypes O, A and Asia 1 using the same VNT method at the same laboratory (WRLFMD) as the current study. These authors considered that the relationship between antibody levels and homologous protection was similar for the three serotypes and different strains analysed at WRLFMD, with a titre of 1.49 being associated with 50% protection. This is approximately mid-way between the titre ranges associated with cross-protection in the present study (Figure 2, Table 2). However, taken as a whole, these homologous potency studies show considerable variation in the VN titres associated with protection including significant differences between some serotypes and strains. The requirement for higher antibody titres for protection against serotype O compared to the levels required for equivalent protection against serotypes A and C has been noted (21, 22), but has not been a universal finding. VN titre differences were also noted when the same sera were analysed against the same virus strains in different laboratories (5, 24).

For registration and batch release of FMD vaccines, potency and immunogenicity trials are usually performed on sera collected from animals that have been vaccinated once, usually 21–28 days previously. However, most FMD vaccine manufacturers recommend that animals being vaccinated for the first time should receive two doses of vaccine, often at an interval of 1 month. Post-vaccination monitoring studies done at population level can be performed in 6–12 month-old animals that have had only the first vaccination. However, when immunity needs to be measured in other age groups this will involve analysis of sera collected from animals at different times after different numbers of vaccinations. It is therefore of interest to know if the same antibody thresholds that predict a certain level of cross-protection after one vaccination would be appropriate after a second vaccination. One of the analysed studies (8) involved challenge of cattle after both a single and double vaccination and this did not appear to influence the correlation between *in vitro* neutralising antibody and *in vivo* protection. However, in the current study we only looked at the titre at the day of challenge in relation to protection in cattle that had been vaccinated 21–28 days earlier. A previous study showed that the relation between antibody titre and protection 9–49 months after vaccination is different (25). This shows that antibodies alone are not responsible for protection but are a correlate of the immune response in the animal.

The experiments analysed in this study used high-potency vaccines, and where tested, some of the vaccines had homologous potency results of >32 PD50/dose (4). This may account for the relatively good protection (65%) seen against challenge strains with a mostly poor antigenic match. These vaccines may be typical of those produced from banks held by FMD-free countries, but lower potency vaccines are often used to control FMD in endemic settings, where cost is a greater constraint.

Since VNT results are poorly reproducible between laboratories, most of the sera were assembled and tested in one place, where the method has been used and standardised over many years under ISO 17025 accreditation, incorporating reference sera, and charting of result trends. Comparing the titres and correlations obtained using results from two different laboratories (Figure 3) confirmed a consistent pattern of differences, that might be eliminated by reference to the results obtained with shared standard sera (26). FMD serology by VNT is mainly carried out by the quality control departments of vaccine producers and by FMD reference laboratories with appropriate biocontainment facilities and procedures. For routine post-vaccination monitoring at population level, commercial ELISAs are recommended for their ease and simplicity but a subset of the tested sera can be sent to a reference laboratory for VNT against specific field viruses of concern (27). Some regions, such as parts of Africa, with a great diversity of strains of FMDV and inconsistent vaccine quality control would benefit considerably from a system of vaccine selection and monitoring that can account for variations in vaccine potency and antigenic match. In response to this challenge, a recent initiative has been the launch of a global prize for vaccine producers who can provide vaccines for East Africa that elicit specific antibody responses measured in terms of VN titres against a panel of representative field viruses^1^. The requirement is for three out of five vaccinated cattle to develop log_10_ 1.5 antibody titres to at least three of the four strains tested per serotype when tested at WRLFMD. This is a pragmatic approach to drive up vaccine standards but carries some risk of excluding adequate vaccines and promoting ones with a low level of protective ability.

In conclusion, testing and analysing day-of-challenge sera from vaccination-and-challenge cross-protection studies, confirms the association between *in vitro* neutralising antibody titre to the challenge viruses and *in vivo* clinical cross-protection. However, different threshold levels of heterologous neutralising antibody were associated with specific levels of protection. This makes it difficult to define serological cut-offs that can predict protection against specific threats with precision. There is a suggestion of higher antibody titres being required for serotype O but other factors influence the thresholds required and remain to be identified. Further vaccination-and-challenge studies are needed to define the thresholds with greater certainty and to better understand what causes them to differ between some studies. Given the difficulty in conducting challenge studies, efforts to collect real-world field data on cross-protection should be encouraged.

## Data availability statement

The original contributions presented in the study are included in the article/Supplementary material, further inquiries can be directed to the corresponding author/s.

## Ethics statement

The findings in this report are derived from analysis of samples or results from already published animal studies, except for three experiments. The two unpublished experiments carried out at Pirbright in 2007 (Table 1, #3) and 2012 (Table 1, #17) were compliant with the Animals (Scientific Procedures) Act 1986, EU Directive 2010/63/EU, and licenced by the Home Office after local ethical review. The unpublished experiment carried out in the animal facility of Wageningen BioVeterinary Research in Lelystad in 2020 (Table 1, #18) was performed according to the Dutch Animal Ethics Law, approved by the Ethical Committee of WBVR-Lelystad.

## Author contributions

DP, DK, AL, and SG: conception. DK and DP: funding. AD, GW, CB, ME, JB, HD, and LP: data provision. SG and DP: analysis and primary authors. All authors: manuscript review. All authors contributed to the article and approved the submitted version.

## Funding

The work of the Pirbright Institute was supported by BBSRC core funding (grants BBS/E/I/00007036 and BBS/E/I/00007037). The study was also supported by grant SE1130 from the Department for Environment, Food, and Rural Affairs and by a WOAH Twinning Grant to the Pirbright Institute.

## Conflict of interest

The authors declare that the research was conducted in the absence of any commercial or financial relationships that could be construed as a potential conflict of interest. The handling Editor declared a past co-authorship with authors DP, AD, AL, ME, and DK.

## Publisher's note

All claims expressed in this article are solely those of the authors and do not necessarily represent those of their affiliated organizations, or those of the publisher, the editors and the reviewers. Any product that may be evaluated in this article, or claim that may be made by its manufacturer, is not guaranteed or endorsed by the publisher.

## Footnotes

^1^ https://www.wrlfmd.org/sites/world/files/quick_media/Cross-neutralisation%20measure%20AgResults%20Final%20v2.1.pdf
