# Supplementary material for: Predicting cross-protection against foot-and-mouth disease virus strains by serology after vaccination
Source: Front Vet Sci. 2022 Dec 1;9:1027006. doi: 10.3389/fvets.2022.1027006 (PMC9751447; doi:10.3389/fvets.2022.1027006)
Supplement: Supplementary file 1 [file Table_1.docx]

**Table S1.** Deviance information criterion (DIC) comparing models for the probability of protection.

| intercept* | slope* | DIC† | | |
| --- | --- | --- | --- | --- |
|  |  | all data | excl. expt 2 | WBVR only‡ |
| common | common | 340.4 | 276.6 | 44.8 |
| common | serotype | 307.2 | 258.7 | 44.9 |
| common | strain | 303.8 | 253.0 | **43.6**¶ |
| common | group** | **286.9** | **235.5** | not fitted |
| serotype | common | 311.9 | 262.9 | 45.1 |
| strain | common | 309.0 | 259.5 | 44.3 |
| serotype | serotype | 307.7 | 259.1 | 45.1 |
| strain | serotype | 306.2 | 257.2 | 44.4 |
| serotype | strain | 306.6 | 254.3 | 43.6 |
| strain | strain | 304.5 | 253.3 | 42.8¶ |

* common: parameter common to all serotypes/strains; serotype: parameter varies amongst serotypes; strain: parameter varies amongst strains

† a model with a lower DIC is preferred to one with higher DIC; the model with its DIC shown in bold is the one preferred

‡ analysis using titres measured at WBVR for experiments 7, 8 & 18

¶ because the change in DIC is less than two and the intercepts did not differ greatly amongts the strains, the model in bold was preferred to the one with the lower DIC

** groups are: (1) experiments 1 & 2; (2) experiments 3, 4, 5, 6, 7, 11 & 18; and (3) 8, 9, 10, 12, 13, 14, 15, 16 & 17; these were defined based on a *post hoc* comparison of estimated slopes in the model in which the intercept was common and the slope varied with strain
