# Supplementary material for: Predicting cross-protection against foot-and-mouth disease virus strains by serology after vaccination
Source: Front Vet Sci. 2022 Dec 1;9:1027006. doi: 10.3389/fvets.2022.1027006 (PMC9751447; doi:10.3389/fvets.2022.1027006)
Supplement: Supplementary file 2 [file Table_2.docx]

**Table S2.** Estimates for the intercepts and slopes in the model for the probablity of protection following challenge with foot-and-mouth disease virus.

| parameter | log_10_ 50% protective titre | | |
| --- | --- | --- | --- |
|  | estimate* | 95% credible limits | |
|  |  | lower | upper |
| intercept (*a*) |  |  |  |
| common to all groups | -3.77 | -5.04 | -2.62 |
| slope (*b*) |  |  |  |
| group 1 (experiments 1 & 2) | 1.98 | 1.35 | 2.68 |
| group 2 (experiments 3, 4, 5, 6, 7, 11 & 18) | 2.92 | 2.07 | 3.88 |
| group 3 (experiments 8, 9, 10, 12, 13, 14, 15, 16 & 17) | 4.16 | 3.04 | 5.46 |

* posterior median
